# The personal motif in naturalistic case study research: developing “innerstandings” in woman’s compulsive behaviour

**DOI:** 10.1080/17482631.2020.1730552

**Published:** 2020-02-23

**Authors:** Tineke A. Abma, Andrea Ruissen, Ellen den Oude, Petra Verdonk

**Affiliations:** a Department Medical Humanities, Amsterdam UMC, Amsterdam, The Netherlands; b Department Psychiatry, Haaglanden MC, The Hague, The Netherlands

**Keywords:** Case study research, person-centred care, hermeneutics, feminist and gender theory, mental healthcare, obsessive compulsive behaviour, women

## Abstract

**Purpose: **The purpose of this article is to show case study research focused on persons as a case and our personal engagement with the case can improve our innerstandings and understanding of person-centred care.

**Method: **We present the methodology and epistemology of naturalistic case study research and illuminate this approach with the case study of Ellen, a young, Dutch, white-middle class woman with a compulsive disorder. We combine naturalistic case study research with the personal narratives of those involved in the research, including ourselves, and interpreted through a feminist and gender lens.

**Results: **The case study research enhanced the personal and mutual understanding of all involved, including the researchers. Feminist and gender theory revealed the hidden personal motif for the choice of the case, and led to a re-viewing of the original story, offering a re-storying.

**Conclusion: **We conclude that the personal motif as well as the use of our personal experiences to understand the case deserve more attention in case study research to address the complex interplay of social and intrapsychic dimensions, and develop more in-depth innerstandings for all engaged.

## Introduction

Person-centred care is increasingly popular in medicine and healthcare. It is a response to the limitations of standardized treatments which are based on the notion that “one size fits all”, or at least many, but which cannot always adequately attend to the varying personal needs of individual patients (Dahlberg, Todres, & Galvin, 2009; Mead & Bower, 2000; Todres et al., 2009; Todres, Galvin&, & Holloway, 2009). Patients vary in terms of sex, gender, age, ethnicity, culture, socio-economic status, and education, and in terms of advantages and disadvantages and experiences of oppression resulting from these social positions, and the complex interplay of these dimensions influences their health, the way in which symptoms are presented, how they respond to treatments as well as patient–provider interactions (Celik, Abma, Widdershoven, & Klinge, 2012; Crenshaw, 1994; Street, 2002; Verdonk, Muntinga, Leyerzapf, & Abma, 2019). Person-centred care responds to the unique personal values and needs of patients, and takes their life-world as a starting point (Dahlberg et al., 2009; Glas, 2019; Hamington, 2018; Mead & Bower, 2000; Todres et al., 2009). This creates methodological challenges. If medical interventions become more tailor-made comparison of their effectiveness via randomized controlled trials becomes more complicated (Greenhalgh, 2005).

Case study research may complement or provide a useful alternative to study the experiences and meaning of person-centred care and tailored interventions. Case studies and case reports have always been popular in medicine (Coles, 1989). Recently, case study *research* is gaining more interest in the medical and health-care sciences (Cronin, 2013; Crowe et al., 2011; Houghton, Casey, Shaw, & Murphy, 2012; Hyett, Kenny, & Dickson-Swift, 2014). Case study research is an approach which tries to understand the complexity and particularity of a case in a real-life context (Abma & Stake, 2014; Simons, 2009; Stake, 2000, 2009). Case studies are increasingly found worthwhile because the methodology allows creativity and flexibility to understand complexity. However, a critical review revealed that case studies are sometimes inconsistent or lack clear justification of the methodology and underlying theory and philosophical traditions (Flyvbjerg, 2006; Harrison, Birks, Franklin, & Mills, 2017; Hyett et al., 2014). Besides the urge to better justify case study research to improve its consistency, rigour, and truth, several authors encourage new ideas for using case study research. Our article wants to show such a new application and is in line with the recommendation to clearly justify one’s epistemological position. Currently, many case studies focus on an organization, ranging from a hospital to a healthcare service unit (Crowe et al., 2011). We want to make a plea for using case studies focused on persons (Abma & Stake, 2014; Røseth, Bongaardt, & Binder, 2011; Tiuraniemi & Korhola, 2009). This person-centeredness of case study research seems to fit nicely with the underlying values of person-centred care.

The purpose of this article is to show how case study research focused on persons as a case and our personal engagement with the case can improve our *innerstandings* and understanding of person-centred care. We combine naturalistic case study research with the narratives of those involved, and another layer of interpretation through feminist and gender theory including the embodied experiences of the researchers. Our case study methodology is grounded in hermeneutics (Kunneman, 2017; Schwandt, 1999; Stake, 2000) and feminist theory (Ahmed, 2017; Butler, 1990; Harding, 1991; Marinucci, 2016). Among the tenets of feminist theory are the embodiment of researchers. Feminist theory assumes that the body of the researcher plays a role in what is found important and whatnot, what is seen and whatnot, what is worthy of research and whatnot. The notion of “innerstanding” refers to knowing from such an embodied, insiders’ perspective. While understandings come from the outside, innerstandings develop from the inside, from those who have lived the experience, and have reflected on that experience. Innerstandings go beyond the rational and incorporate feeling and emotion (see also: Hartman McGilley, 2009; Kimura, 2004). Feminist theory urges researchers to situate themselves and reflect on the personal motifs for the selection of the case.

This article starts with presenting the theoretical position of our case study research approach, subsequently, we will illustrate the use of naturalistic case study research for personalized care with a case study followed by a re-storying of the patient’s story through feminist and gender theory, a personal reflexive layer from the researchers and a discussion on the synergy between person-centred care and the personal motif in case study research.

## Case study research and the naturalistic approach

Case study research aims to understand the complexity of a demarcated entity or case (Flyvbjerg, 2001; Harrison et al., 2017; Yin, 1994). We are interested in people in specific circumstances; a patient, a family, a nurse, or a medical doctor and concentrate on the particularities and uniqueness of a person as a case. Our naturalistic approach to case study research is grounded in interpretivism (Gadamer, 1960), and our objective is to grasp the meaning of experiences through dialogical understanding. In dialogue object/known and subject/knower mutually interact and influence each other in their search for meaning and shared understanding. In line with naturalistic case studies we work towards hermeneutic understanding (Abma & Stake, 2014; Stake, 2000, 2006, 2009), and we explicitly combine this by bringing in our own personal experiences as well as our various scientific and professional fields of expertise. Also, in line with the transformational purposes of feminism and participatory action research, our aim is not just to understand the world, but to heighten the personal and mutual understanding of those whose life and work are at stake (Abma et al., 2019).

The selection of a case in naturalistic case study research is guided by the principle of “the opportunity to learn” (Abma & Stake, 2001, 2014); these cases are often exceptional and revealing complexity, and do not need to be representative to learn and generate knowledge from it (Flyvbjerg, 2011; Stake, 2000). We will show how we discovered later that the case selection was entangled with our own experiences as women; the case spoke to us because it reflected our own struggles and desires.

The design of naturalistic case study research is not preset or structured by a causal theoretical model or hypothesis to be tested, but follows the emic issues of the case. This is not to say that the case is approached empty-headed. As in other qualitative research, the position of the researcher is important in “what is seen” and whatnot, how s/he looks and what is noticed (Entman, 1993; Harding, 1991). Critical reflexivity is, therefore, part of naturalistic case study research in order to be transparent about one’s subject position and relational dynamics between researcher and researched (Råheim et al., 2016). Yet certain things are hard to see when we are untrained, when we are silenced about aspects of the self (e.g., gender issues such as the expression of gendered emotions or experiences), when we are trained into perceiving phenomena a certain way or into not perceiving phenomena (doing gender) (Ahmed, 2017; Butler, 1990). Therefore, it is decisive that researchers learn about themselves, show reflexivity of their own position, and dare to ask questions about their own positions (Verdonk, 2015). The “open-mindedness” required for naturalistic case study also needs to be nourished (and triangulation is needed) by other (theoretical) perspectives. Finding the right balance between being open and responsive to emerging issues and having some initial sensitivity to what is important requires artistry which can be compared to clinical wisdom (Denzin, 1994; Eisner, 1985).

Naturalistic case study research is performed in an ordinary setting of the case, and there is a preference for methods and procedures that are natural, such as storytelling, conversations, and observations which require a “socio-anthropological” sensitivity (Stake, 2000). Interpreting a particular case means that the researcher constructs a meaningful narrative that weaves all the elements and multiple perspectives of the case together (Bray, 2019; Schwandt, 1999; Sools, 2013). The variety of perspectives will make the understanding of the case richer and more informed (Guba & Lincoln, 1989; Jackson & Mazzei, 2013). This implies that one strives for a contextual interpretation of the case and that one is not satisfied with a list of unrelated, decontextualized issues (Simons, 2009). Naturalistic case study research provides local knowledge that is time and context bound. Abma and Stake (2001) speak of a “naturalistic generalization” when those reading the case study report translate the experience from the studied case to their own context. Readers will be better able to do so if the researcher provides “thick descriptions” of the case (Geertz, 1973). A thick description reveals participants’ meanings and context, their unique way of seeing and saying things, and offers nuance and detail. As Robert Stake notices: “a small aspect of the case may be found by many readers to modify an existing understanding about cases in general, even when the case is not typical” (Stake, 2000, p. 443). Over the course of more case studies of a same phenomenon, patterns and “petite” generalizations can emerge (Abma & Stake, 2001).

Relevant quality criteria in our approach to naturalistic case study research are derived from the work of Guba and Lincoln (1989) who introduced credibility criteria (Cronin, 2013; Houghton et al., 2012), and authenticity criteria. The authenticity criteria are particularly relevant for us because these reflect our purpose to heighten the personal and mutual understanding of those engaged in the study. Understanding one’s situation may lead to empowerment as well as an increased capacity to change one’s situation (Abma et al., 2019).

## From studying the illness to studying the person with illness

The case study research presented here was part of a study that aimed to develop a notion of competence in patients with obsessive compulsive disorder (OCD). Competence in patients is defined as the ability to make treatment decisions (Ruissen, 2015). A patient with OCD is generally considered as being competent without explicit assessment.

### Clinical theory on obsessive-compulsive disorder

Obsessive-compulsive disorder (OCD) is characterized by repetitive thoughts with unwanted content (obsessions) and rituals, such as washing, cleaning, checking (compulsions). Obsessions invoke anxiety, while compulsions aim to alleviate the anxiety caused by obsessions. Often OCD is understood as an anxiety disorder. Patients with OCD spend excessive amounts of time with ruminating, worrying, performing rituals, often several hours a day, but sometimes up to more than 8 to 10 h a day. Often they feel ashamed and are hiding their symptoms for their family, friends, and for their mental health-care professionals (Ruissen, 2015). Not all patients experience this anxiety even explicitly, especially not in the group with poor insight. The DSM-classification of OCD has a specifier for this poor insight. The question if poor insight implies incompetence and how competence and insight are connected is ethically important and relevant for daily care. A systematic review of the literature showed that both adequate insight and poor insight in OCD can be associated with incompetence (Ruissen, Widdershoven, Meynen, Abma, & van Balkom, 2012).

In the Netherlands, OCD-patients are treated most often in outpatient clinics for anxiety disorders and OCD. There are general outpatient clinics and academic outpatient clinics. Next to that, there are a few daycare facilities and clinical facilities with psychotherapeutic and psychiatric treatment options.

### Plugging-in a feminist reading: revisiting the story

In an earlier study two of us (AR and TA) conducted, the question arose how patients and therapists actually understand competence in their clinical practice. After collecting experiences from patients and psychiatrists in 18 cases, the particular case of Ellen (pseudonym) stood out (Ruissen, 2015; Ruissen, Abma, van Balkom, Meynen, & Widdershoven, 2014). The case was that of a young female medical student with a compulsive disorder who underwent treatment twice. Her insight was adequate, with average prognosis and a serious burden of disease. Atypical aspects were that Ellen was medically trained, familiar with the concept of competence, and that although initially she refused treatment later she did consent. This combination of typical and atypical elements made this case worth studying: it was embedded in regular psychiatric practice, and nevertheless so special that it required further study.

A literature study on competence and competence assessment from a medical and philosophical framework helped to foreshadow the problem to be studied: besides cognitive capability are there other aspects of competence described in the literature? The case study research included the perspectives of the patient and therapist (pseudonym Susan) and was grounded in two semi-structured in-depth individual interviews with both, in 2014. The interview style was open and similar to a natural conversation to elicit the lived experience (Reissman, 1993). The interview transcripts were analysed using narrative analysis (Lieblich, Tuval-Mashiach, & Zilber, 1998; Ruissen et al., 2014; Sools, 2013). The emic issues were initially related to the concept of “practical rationality” (Author). In between 2010–2015 the findings of the case study were discussed with the participants (Ellen and Susan) to check the credibility of interpretations. Both agreed with the interpretation and reflection, and approved the first publication of their case report (Author). Later, another layer of interpretation was added using feminist and gender theory and our own embodied experiences to gain richer insights into how the social position of Ellen played a role in the development of compulsive behaviour.

In the earlier study, two of us coded the interviews (AR and TA) and themes (emotions and values) emerged from the data were related to the concept of “practical rationality” (Ruissen et al., 2014). In this paper, we revisited the initial interpretation of Ellen’s story and from a feminist perspective. The coding and interpretation was done by three of us (AR, TA, and PV), and in a later stage the participant also got engaged in the renewed interpretation of her story. We think that feminist analyses are insightful in Ellen’s case because the feminist lens offers new understandings of the case (Harding, 1991). It is comparable to what Jackson and Mazzei (2013, p. 261) characterize as a strategy of “thinking with theory”: they compare the process of analysis and interpretation of interviews (texts) with an assemblage of plugging-in one “text (machine)” into another text (machine) in order to produce new, interesting understandings. They see this as a way to counter well-rehearsed notions and find alternative understandings that take into account tensions and ambiguities.

We added the epistemology of Ellen as a “legitimate knower” herself. This was given in by what Miranda Fricker (2007) has described as a strategy of “epistemic justice”: to actively include the voices of those whose issues are at stake, and who have formerly been wronged in their capacity of knowing (for example, women), and whose voice did not count as relevant. Furthermore, we brought in personal selves as “agents of knowledge” beyond our distanced, academic, professional gaze, because personal, embodied experiences are important resources of knowledge (Ahmed, 2017). In such an epistemology, women’s and marginalized voices are important, for instance, because their questions may point towards different phenomena. In the case of Ellen, we directed our gaze not towards her expressions of competence or possible lack thereof, but rather, to the story itself: the fear of death, the sense of (un)safety, the self-silencing after the traumatic experience in her youth, the shame and the struggle, and we revisited that story by aiming to make sense of her desires and experiences rather than to discuss the irrationality of her behaviours and her aims to control them. Hence, we looked at what problem Ellen aimed to resolve with her behaviour, even when this also created problems in her life—we understood them as meaningful and telling. As such, we tried to deepen our understanding of the problem behind the problem as defined in psychiatric, diagnostic terms, and rephrase it. Therefore, from a feminist perspective, we generated the problematics in this case from Ellen’s and our own personal experiences. By doing so, we also placed ourselves under scrutiny as well, instead of presenting ourselves as the anonymous voice of knowledge (Harding, 1991; Ellis, 2004). This also meant that we developed a more horizontal and equal relationship with Ellen, and all became involved in studying our lives (Abma et al., 2019).

This required a re-reading and analysis of the case study and the original transcripts. While initially the analysis had seemingly been “neutral” and zoomed in on Ellen’s individual experiences and understanding, a gender and feminist re-reading zoomed out on Ellen’s social position and how this position interfered with intrapsychic dynamics, and on questions like what does it mean to grow up as the oldest daughter of parents with relation problems, what does it mean to be a girl in Dutch society, how does Ellen deal with her first sexual encounter and what does it mean to be enrolled in medical education? This secondary analysis and interpretation generated new themes amongst which stood out (a) the recognition of Ellen’s fear of death just after her first and negative sexual experience and, (b) the benefits of seeing a regression therapist who helped her to feel her pain and develop more compassion with herself. This analysis and interpretation included a series of intense, reflexive dialogues among the research team; face-to-face, by telephone and email, but also reading each other’s literature and taking into consideration relevant discussions, for example, on women’s sexuality and neglected trauma in psychiatry. These aspects of the case required an in-depth communication because our disciplinary perspectives on it were quite different. Although we agreed on a general level that a biopsychosocial model was helpful to understand Ellen’s OCD, we were not sure whether the OCD was related to sexual trauma (feminist reading) or a crucial life event and outcome of psychodynamics in the family (clinical reading), or both. We arranged a second interview with Ellen in 2018 and a third encounter in 2019 to deepen and validate our understandings. A participatory member check led to a deeper discussion on the key themes and fostered co-ownership of Ellen (Doyle, 2007).

This process led to a “re-storying” of the case (Brown, 2013). This “re-storying” is in line with the narrative theory that stories are the outcome of a hermeneutic and dialogical process, and part of an ongoing negotiation (Walker, 1989). Ellen’s story should thus not be considered finished. Publishing their story (again) can be seen as a continuation of the narrative construction process.

The study was approved by the VUmc Medical Ethical Committee. Participants gave written informed consent. Anonymity was assured by using fictitious names. Case study research written as a story encounters several ethical tensions. One needs, for example, to be careful not to solidify the case in a way that hurts those involved and take the future into account (Plummer, 2001): case study research is intricate and personal. We gave Ellen contact addresses of the researcher in case after-care was needed, and a few weeks after interviewing we contacted her to ask how she was doing and how she looked back on the interviews. Both Ellen and Susan experienced participation as empowering; the study gave them new insights for their own life and practice (see also Ruissen et al., 2014). For the secondary study again permission was asked for publication from Ellen and approved by her. We also asked her to become our co-author to give her credits for sharing this intimate story, which she accepted but under a pseudonym to protect her privacy and confidentiality. The stigma also withheld her to publish under her own name. After reading the draft version of the article she emphasized the “therapeutic value” of the contextualized interpretation of her story, stressing the de-stigmatizing effect it had on her:

Most remarkable was the resonance and recognition (with some aspects of my social position). That was touching. A special experience … It softened the stigma.

## The case: Ellen, a patient with obsessive compulsive disorder

Ellen was a Dutch, white, female, middle-class 30-year-old medical resident, diagnosed with OCD 10 years ago, still being a medical student. Her symptoms started in puberty. She was afraid of infection, primarily human immunodeficiency virus (HIV). Initially, she was able to live with her fears. During medical education, Ellen lived with her boyfriend in the basement of her parents’ villa. The relationship with her parents was quite rational. They supported her, but she felt they did not really understand the depth of her suffering and could not always provide emotional support. Hence, Ellen mainly relied on her wider social network. To stay in control, she often visited an HIV clinic anonymously asking for blood tests to receive assurance that she was not infected. A few years into her medical training, her symptoms worsened significantly. She was referred to a local male psychiatrist who believed she was suffering from some kind of medical-training-related hypochondria. Unhappy with his approach and feeling that she was not able to profit from this treatment, Ellen had herself referred to an academic centre, where she met a second male psychiatrist, who referred her to a behavioural therapist, Susan. They embarked on behavioural therapy preceded by a motivational approach, including a family session. Later, Ellen went to see a female regression therapist. After the treatments, Ellen felt much stronger than in the period before the compulsive behaviour developed, but she said she would never forget the symptoms she had. Currently, she is living with her husband and daughter, and works as a clinician.

Below we present Ellen’s story. The story of Susan has been published in the earlier version of the case study, and will not be presented again because it had not been altered in the secondary analysis from a gender and feminist perspective.

### Ellen’s story

Ellen thought of herself as a “perfectionist”: “Always wanting to do things right, everything done in time, could be very restless (…) [but] it wasn’t a problem”. She heard about HIV-aids when she was 13. She considered herself as having mild obsession and compulsion since she was young, as lots of children have. In retrospect, she notices that more serious obsessive-compulsive symptoms began after an “unpleasant sexual experience” with a boy during a holiday abroad. It was not the experience per se, but the thought of the possibility that he was infected that scared her enormously. She talked in terms of an existential fear:

I remember … he was kind of small, and I was with my girlfriend. So, nothing to be worried about. And yet, I felt scared to death afterwards … it triggered a fear of death inside me.

She panicked, a panic she could not share with her parents because she felt ashamed and feared their disapproval. All she could think was “they are going to be mad if I am too late”. Back home again she had herself tested for HIV. Years later the urge to get tested came back. There was one particular moment when her symptoms became really troublesome:

So the solution was to start washing—hand washing, hair washing, cleaning the house, washing clothes, showering, and avoidance. I could do that to some extent but I told myself I had to go to [university]. I had to finish my education, those were two things that I had to do. (…) I couldn’t stand it that I got carried away with it. I was also angry (…) I was very angry with myself. (…) I got carried away by my thoughts (…) I couldn’t stand it, it was just like an addiction. I couldn’t stand the idea that I had to give in to it again, because it just didn’t work.

Aware of her symptoms, Ellen did consider herself competent though. She believed she was able to reason and understand what was happening. In her opinion, her cognitive abilities made her competent:

I could always justify why I did things. I knew exactly why I did it. Though it was with a thousand detours, I could always reason very well. (…) Because I felt the urge to do something, I tried to make things right, and that is what I did.

Ellen “knew” very well what she did and could “reason” about her compulsions, and make “things right” by combining obsessions, compulsions, and real life. Her cognitive functions were not disturbed. However, her feelings and intuition were more of a problem to her. She experienced her obsessions and compulsions as “not normal” and agreed to be referred to a psychiatrist. Nevertheless, she was unable to commit herself to treatment and did not benefit from the care. And although she concealed her major symptoms from her first psychiatrist, she still thought of herself as being competent. The second male psychiatrist changed her attitude towards treatment and occasioned a turning point:

Then he [psychiatrist] asked me, ‘what do you want’. And actually, I always had my doubts about that question. But, in this context it was really important. (…) Very important, crucial. (…) I was forced to show how I should get better. (…) I said: yes, I will do everything I’m afraid of. Otherwise it won’t go away.

The psychiatrist’s stance, on urging her to explicitly state what she wanted, led her to feel she was being recognized. Ellen felt not recognized before, not with the parents, not with herself maybe—and now, someone actually saw her. We can see the motivational approach, starting with what Ellen wanted, stimulating her intrinsic motivation to start treatment. It then became clear for her that finalizing her medical education and getting back her life, were the main cues to action to get rid of her obsession to control her fear. So she accepted the referral to behavioural therapist Susan. Based on her medical knowledge, Ellen “knew” what the best intervention was: to expose herself to anxiety evoking situations and refrain from anxiety-reducing rituals. But she was also scared and felt she was “working against” herself. The change in attitude from avoiding to engaging in therapy was hard for Ellen. She did commit to this treatment, but this commitment came at a price: anxiety. She was, nevertheless, highly motivated. One important value—finishing medical school—was still an important incentive for her:

I wanted to do a lot, because it [OCD] had to end soon. Because I wanted to finish my education. (…).

After the treatment Ellen still did not understand her vulnerability:

I felt I wasn’t bullet proof. I wanted to understand.

Thus, she went to see another female regression therapist who followed an embodied and affective approach. She remembers crying and initially not understanding why. Showing her feelings felt strange:

She was very down to earth . and I had to lie down on a sofa, close my eyes, and then I came into a trance, hypnosis or something, and had to tell her what was wrong. And what I felt, and where I had that feeling. Well I thought, that is weird, and I said that to her. But meanwhile I was lying there. On that couch, crying out, saying all kinds of things, of which I think, what are you saying right now, what do I say. And then she said that doesn’t matter, just say what comes to your mind.

Although it felt awkward, the regression therapist deepened her understanding:

She [the regression therapist] touched another, deeper layer … helped me to look at myself from a distance … to see the little girl, to cherish [that girl] … there is a huge vulnerability inside me … I know that, and still find it difficult to acknowledge that. There is still that resistance.

Gradually Ellen developed more compassion with herself and her vulnerability, and she came to a better understanding of her relationship with her parents through expression, articulation, and inquiry of her feelings. She began to realize why she did not like her parents telling her she had to stop reading and studying. Their concern that she was overambitious and not relaxed felt not empathic but rather quite instrumental as if she did not matter. She felt that her parents did not touch on the reasons why Ellen wanted to study so hard and did not understand her need for them to be proud of her:

My parents said, ‘Do something fun and enjoyable’, put your book away. This irritated me enormously, I thought, well, be proud.

Ellen also began to realize how intrusive the alleged divorce of her parents was for her, how responsible she had felt for their marriage, even guilty, and how this had steered attention away from her to their relationship problems. Through an inquiry of her feelings Ellen changed. When asked, Ellen reflected on the change:

Well I’m very different. I think. Especially that perfectionism, and overprotectiveness, that’s gone. I live more day by day, I would say as a cliché. Of course I worry about lots of things, but I don’t think any more than the average person does, actually. (…) I’ve learned to put things into perspective very well. That’s well developed. (…) I do a lot on intuition. So I basically live mainly from intuition. And if things get a bit out of hand, then I see what’s going on. I’m not that easy to unsettle, at least far less than before.

Perfectionism was a character trait she had had since she was young. But she was now able to loosen up. She now spoke in terms of “I live by the day” and “I live intuitively”, and talked about relationships and her character as temperamental but relaxed. She became more flexible because there may be wants every single day, different wants and needs, and they need to be taken care of when they arrive. Instead of continuous “control” over her life through compulsions, she was able to regard her life when things got out of hand. She was not easily put out of balance. Ellen mentioned more changes:

I know why it went wrong, and that’s when I was avoiding everything, when I was denying it. And that I was trying to shy away from it, whilst I had to confront myself. When I cycled back once in a while, I found that terrible of myself, but I can forgive myself too, because I know that it may be worse today than tomorrow. (…) That insight has grown, but it was already there. I knew it wasn’t normal.

Interestingly Ellen still thought in terms of fear and failure when she talked about “forgiving” herself. Here we can see that making mistakes was still something that was not allowed; having compulsive thoughts was still not “right”, still a transgression, yet “forgivable”. Yet, at the same time, Ellen was more willing to “admit to her vulnerability”, she no longer had to hide or negate it. She knew where her vulnerability lay: in her tendency to want to control things, particularly when feeling tired and unsettled, particularly at home. She could better accept her irrationality and fragility and felt more able to trust and rely more on her senses and intuition. Ellen was even able to put one of her main goals and values in life, i.e.,, finishing her medical studies, into perspective. This was quite a radical change for her. A final change was that Ellen got less preoccupied with herself.

When asked about the meaning of this episode, she admitted it stood for much more than getting cured:

Well, yes, it actually enriched my life.

These changes have also brought about a different idea of what it means to be competent. Initially, for Ellen, being competent meant being able to reason, knowing why she performed the compulsions and recognizing that they represented suffering. Later she began to understand that being competent had a lot more to do with emotion and value, and with being able to live an authentic life, knowing herself.

Below we share our second reading inspired by feminist and gender theories.

## Another perspective: looking through a feminist lens

The case reveals that Ellen’s compulsive behaviour was related to the experience of not being able to share a negative sexual experience with her parents, resulting in distress for two reasons. First, she was not being recognized as a (sexually active) girl and not being recognized as a child in a quest for identity and psychological growth including fears and emotions. And second, she felt unsafe because of the potential divorce of her parents and the appeal on (her) responsibility. Cromer, Schmindt, and Murphy (2006) describe how painful experiences can be related to developing compulsive behaviour; the mechanism where intrusive, traumatic thoughts are controlled by certain actions, such as hand washing. In Ellen’s case, the negative sexual experience was a transgression of her boundaries, which led to feelings of disgust. It appears as an ignored but crucial life event. Ellen considered herself responsible for it and took the blame, a well-known pattern (Brown, 2013), often grounded in a “good girl script”, which may give a sense of control over the situation. The “good girl script” (Ahmed, 2017; Butler, 1990) calls among other things for a withholding, a toning down, a tucking in of expression, or even complete silencing (Jack & Ali, 2010).

The case study thereby illuminates the relation between compulsive behaviour and Ellen’s social position. The onset of the compulsive behaviour was a gender issue, as was her response. Especially adolescent girls find it hard to deal with social norms on feminity and sexuality, and may give in to men (Smolak & Murnen, 2002; Worell & Todd, 1996). Moreover, sexual life events are experienced differently by men and women, and usually have a greater impact on girls and women (Smolak & Murnen, 2002; Worell & Todd, 1996). Women are typically expected to have induced the harassment. In Ellen’s case, she silenced herself (“it was not against my will” and “I couldn’t share it with anyone”) starting a process of more and more symptoms over time (Jack & Ali, 2010; Yoon, Funk, & Kropf, 2010). Ellen’s response is understandable given the ambivalent social norms on girl’s sexuality. Girls receive contradictory messages when it comes to their sexuality. They are expected to present as sexually desirable, but simultaneously need to be cautious to not overstep boundaries into “sluttiness.” This requires self-monitoring which can lead to alienation, disembodiment, and lack of connection in sexual encounters and relationships (Tolman, 2018). Research indicates this can result in lacking a sense of entitlement to one’s own body, feelings, and the right to really say “yes” or “no” and be respected. In that context, we can understand how Ellen “performed” her sexuality, and was not able to feel and express her boundaries. The experience must have been unsafe and transgressing, otherwise, Ellen would not have such strong feelings of disgust and shame afterwards (Dykshoorn, 2014); a shame and a disgust to the self which were almost self-undermining (Price Tangney & Dearing, 2002). She was not accidentally afraid of infections, and more particularly of HIV as a Sexually Transmitted Disease (STD).

Besides the self-silencing and consensual ideologies about sexual adverse events, her perfectionism/compulsion was also gendered as well as her insecurity. Ellen tried to overcome and control her emotional pain through socially desirable behaviour. She wanted her parents to be proud of her, worked hard for good grades, but experienced that her parents were overloaded with their own relational problems. She felt responsible for the happiness of her parents, which is typically a gendered role endowed to women (Worell & Todd, 1996). Besides, Ellen was wrestling with the distinction between being worthy as a person and worthy for what you accomplish. Price Tangney and Dearing (2002) make exactly this distinction between shame and guilt. Shame is what you feel when you *are* wrong, guilt is what you feel when you *did* something wrong. In Dutch society, girls develop less self-confidence than boys, and women feel less secure than men. Women and men are socialized into the belief that women are not as competent, intelligent, capable as men (Nolen-Hoeksema & Jackson, 2001; Nolen-Hoeksema & Larson, 1999). They constantly feel the need to work hard, to be perfectionistic to overcome the feeling of not being good enough, which in itself can lead to stress, anxiety, and depression (Verdonk, de Rijk, Klinge, & de Vries, 2008). The idea that one should be perfect and autonomous is also part of the white Western meritocratic culture and was in Ellen further triggered by her medical education with a strong cognitive orientation and focus on being empathic but stoic and detached, not paying attention to one’s own feelings and vulnerability (Bleakley, 2013; Verdonk, Räntzsch, de Vries&, & Houkes, 2014).

Ellen’s response to treatment is also gendered. Initially, she would “replay” the experience rather than “process” it (Van der Kolk, 2016a). The cognitive behavioural therapy helped to develop new behaviour and rationally understand what happened, but it did not and cannot *undo* emotions, feelings, or thoughts (Van der Kolk, 2016a). Emotions need to be empathetically felt and soothed, like in the regression therapy focused on the lived body (Hartman McGilley, 2009). Ellen needed the regression therapist to understand and cherish the “little girl” inside her and work out the bodily and emotional aspects of the life events—yet in less controlling and disciplining and more accepting and self-compassionate ways (Hartman McGilley, 2009; Smolak & Murnen, 2004). A process that induced more self-compassion, but not complete acceptance (“There is still that resistance”). We can now also see why it is only possible to be competent when we are connected to our own emotional inner-life. If competence is understood cognitively, as it usually is defined, this does not work for people like Ellen who cognitively knew that it was important to set boundaries, but who could not feel her boundaries because there was no contact with inner emotional life. Ellen benefited from regression therapy to develop an “*inner*standing” (Hartman McGilley, 2009) and to feel she was allowed to be vulnerable and that not everything can be controlled (Johnson, 2008). Perceiving what happens inside us, and therefore being able to feel is required to reconcile with our emotions. Such a sense of the internal state of the body is called interoception (Van der Kolk, 2016a, 2016b).

Finally, we can see how Ellen’s autonomy developed in relation to others’ interference, like the first therapist who asked her what she wanted. This is an important aspect of female socialization, not being asked what you want, your wants being silenced, and at some point, not even knowing what you want when they explicitly ask (Ahmed, 2017). When being asked what she wanted, Ellen began to matter. The second therapist elaborated on this, starting from her wants and needs. Developing one’s identity and voice with others is in line with feminist approaches to therapy (Hartman McGilley, 2009) and care ethics in which autonomy is conceptualized as the ability to shape one’s life according to one’s values in relation with others (Tronto, 1993).

## Our own embodied and learning experiences

In retrospect, the case was also particularly interesting to us because we could identify with Ellen. Stories are not neutral, they touch us because they link up with our own experiences. As Arthur Frank (2010) notices, based on his concept of “narrative habitus” people are predisposed to pay attention to some stories and not to others. In our case, the stories were relevant given our social positions as senior female academics in the field of medical humanities (first author) and gender studies (last author), and the second author being a medical doctor herself working on her PhD at the time. The case offered us the opportunity to make the personal political. Revisiting this story invited us to share more personal experiences with each other, to re-evaluate these experiences and hence, start seeing them in a different light. For instance, one of us immediately talked to her parents after an incident of harassment, after which the parents took measures that were logical, and fair. This response helped to re-establish a sense of safety, and although the incident was not further addressed, the silence was not a sign of (self-)silencing. We recognize the following:

- The self-silencing and taking responsibility for the well-being of others and not of ourselves;
- Academics with a high need for achievement, and control in order to succeed, be seen and acknowledged;
- Experiences of sexual harassment (one of us also had a positive experience afterwards, went to see her mother who immediately intervened);
- Rational parenting styles and attachment distress (e.g., with mothers and fathers who could not flourish due to adherence to the gender norms at that time) (see., e.g.,, Van der Kolk, 2016a, 2016b);
- Focus on cognition at the expense of spirituality and sacredness (Hartman McGilley, 2009), love and embodied intuition (see also Bleakley, 2013);
- Self-silencing in the form of initially not seeing the gender perspective (Brown, 2013); blind spots and taken-for-granted patterns because we are in the middle of it as researchers;
- A focus on the individual, autonomous self as a major tenet of Western norms, as expressed in self-reliance and independence.

## Contribution to and congruence with person-centred care

The strength of naturalistic case study research lies in its rich and multi-layered understanding of the singularity of a single case, using multiple reality constructions in a particular time-location-culture context. The case presented revealed the interactions between patient and therapist, and both perspectives enrich our understanding of how decisions are made. A second layer of interpretation through a feminist lens opened up new vistas on the social position of the patient and further enriched the interpretation. New issues came up such as the ambivalence in Ellen’s story, the relation between painful experiences and compulsive behaviour, the self-blame and self-silencing and shame hidden under the façade of perfectionism, fear of failure and secrecy. It also shows the interactive dynamic and hermeneutic process of naturalistic case study: both participants were invited several times in the research process to share their stories as well as to check the credibility of the interpretation. Then, a process of re-storying began by introducing a feminist perspective and the embodied experiences of the researchers themselves.

The case study research enhanced the personal and mutual understanding of Ellen and Susan, and this was further intensified after the second feminist reading including Ellen, and the authors. For Ellen, the interviews resulted in more personal insight in her process of growth and her quest for the right treatment, which was a parallel process with her quest for more competence and better decision-making. Looking back on that process made her even more convinced of her decisions. She felt empowered by being interviewed and emphasized its “therapeutic value”: “It is hard to reflect on your own”. The retelling of her story, not only in terms of grievance but including elements of resilience and personal growth was important for this empowerment (Charon, 2001). This process was stimulated by an empathic interviewer, who listened to the story, who probed, and who helped through deliberation to develop a new understanding of the whole process with an eye for resilience (Brown, 2010). Susan recalled it was helpful for her to realize that her motivational approach and attitude were apparently adequate and resulted not only in symptom remission but also in good care as defined years later by Ellen. Her intuitive way of working could be grounded in the tradition of deliberation (Emanuel & Emanuel, 1992). Susan felt empowered because she now understood that her way of working is value based. Through the process, both women came to a shared understanding of the importance of other than cognitive capabilities for decision-making and recovery. As pointed out the gendered analysis of the case spurred an inquiry into our own personal lives as researchers, and fostered emotional recognition, awareness of our position in society, and sense of belonging. This process of understanding is hard to realize with more common qualitative strategies such as interviews or focus group studies.

Naturalistic case study research is synergistic with person-centred care. The focus from person-centred care is on the patient as a person with unique needs, values, and life-world (Dahlberg et al., 2009; ; Hamington, 2018; Mead & Bower, 2000; Todres et al., 2009). In person-centred care, patients’ experiences and needs are not reduced to a set of signs and symptoms within a biomedical framework (Glas, 2019; Mead & Bower, 2000). There is an acknowledgement that patients’ symptoms are not purely biomedical (Hoffman, 2000), but influenced by their social position, like in Ellen’s case her gender, Dutch middle-class culture and medical school context with its meritocratic, cognitive orientation that even more triggered her perfectionism and need for control. The case study shifts the focus from the particularities of the illness to the particularities of the person with an illness. This opens up new perspectives and patterns: instead of illness patterns, the case study reveals underlying dominant discourses of being in the world—as a girl and a victim-in-control (Brown, 2013; Verdonk et al., 2008), as a patient (Dykshoorn, 2014), as a medical student (Bleakley, 2013; Verdonk et al., 2014).

Person-centred care pays attention to the special needs of an individual patient following from the interplay between sex, gender, ethnicity, class, and other dimensions of difference (Verdonk et al., 2019). Time is invested in patients so that they feel respected and acknowledged as persons. The personal values, opinions, and needs of patients are taken into account (Glas, 2019). In the case, we saw that for Ellen it was crucial to rebalance, to develop more confidence in herself, to let go her need for control to hide from fear to “admit to vulnerability” (Brown, 2010). This person-centred approach resonates with the focus of naturalistic case study research on the patient as a case, and the particularities of a case and the holistic, contextual understanding it generates. The move from single causes to multiple, mutually interacting factors and the need for coordination from different angles in person-centred care reflects naturalistic case study research embrace of contextual interaction, mutually shaping forces, and complex webs of influence in human life and health. This shift from particularities of the illness to particularities of the person with an illness might offer new interventions; ones that are not necessarily targeted at the particular illness, but possibly to patients at similar crossroads of their social positions and beyond (e.g., Verdonk et al., 2019). For example a group intervention for young women with comparable control-not-being-vulnerable obsessions, yet different diagnoses, based on feminist principles (Hartman McGilley, 2009).

We can also see a parallel between naturalistic case study research and shared decision-making. In healthcare, there is a move from passive constructions of health to active and meaningful participation in the diagnostic process, in setting up and tailoring interventions, from the absence of disease to positive health, resilience, and wellness (Antonovsky, 1996; Brown, 2010). Also, we see a professional posture where responsibility is taken for a whole patient and shared equally between practitioner and patient (Glas, 2019). In the case, we saw that Ellen wanted to understand her illness, and that the dialogue with her therapist helped her in the process of meaning-making as well as the retelling of her story during the interviews. The therapist relied on a motivational approach, engaging Ellen in the decision-making process. The therapist paid attention to the family, and acknowledgement of the social context is considered an important aspect in gender-sensitive care: good care cannot do without paying attention to the structural position of patients (and caregivers) and their web of relations (Verdonk et al., 2019). In the relationship patient and therapist were not superior or inferior to one another. The fact that the therapist was also a woman and medically socialized helped in the process of understanding; it is easier to feel empathy for someone in a similar social position. The regression therapist was also a woman, but less rational and cognitive, and therefore able to “repair” the broken contact with Ellen’s emotional inner life.

Meaning was created in the encounter and ethical-relational space (Abma, 2005; Pollard, 2015). This is especially important in attachment issues, as seem to occur in Ellen’s case. Attachment, including in shame, can only be restored in relation with others (Van der Kolk, 2016ab). For shame, a deeply relational emotion thrives in secrecy, and hence, talking about shame, admitting to vulnerability, can only take place in an empathic relation between self and others (Brown, 2013; Price Tangney & Dearing, 2002). During the process of interpersonal understanding, new themes and questions arose that directed the treatment process. This process reflects the move to shared decisions, shared constructions and dialogue in naturalistic case study research (versus mere object of study).

## Discussion

The most important characteristics of naturalistic case study research are its holistic focus on one case to understand its complexity from multiple perspectives through time. Case studies of persons can reveal emotional and cognitive changes (Røseth et al., 2011; Tiuraniemi & Korhola, 2009), and generate context-bound knowledge. The methodology is flexible, emergent, and iterative leading to “emic” issues (Harrison et al., 2017; Hyett et al., 2014). In naturalistic case study research, a substantial theory is not the starting point but is brought in later in the process to deepen the emic issues, in line with the “thinking with theory” strategy of Jackson and Mazzei (2013). It builds on and uses personal experiences of the researchers and participants as important resources of knowledge to counter epistemic injustice (Fricker, 2007) and generates interpretive understandings of what a meaningful, and fulfilled life entails, and can lead to a re-storying of the case. Personal engagement with the case and interpretation through a feminist lens opened up new themes including the self-silencing of traumatic sexual experiences, the complicated relationship with parents and disciplining nature of the medical school. This created some discomfort among the authors, because as soon as we began to see the similarities between the case and ourselves it felt like putting ourselves under a magnifying glass by studying ourselves, which is also felt in auto-ethnography (Denzin, 1994; Ellis, 2004). We were surprised that the gendered nature of this case was initially overlooked, partly because we were not trained to see gender issues.

In retrospect, we appreciate how the case was not only chosen on the basis of rational criteria but also because we could personally identify with the case (Frank, 2010). This personal motif was initially overlooked, and not explicitly articulated. We felt intuitively the case mattered, as we recognized Ellen’s individual struggle from being in a similar social position (white, middle class, females, highly educated, and working within healthcare). Later on, we explicitly reflected on our own embodied experiences and recognized how we feel the need for control and how we are wired to ignore our vulnerability. We did not have the OCD label, but recognized underlying patterns in our lives and work. So as researchers we interacted intensively with the case on a personal level to come to a meaningful understanding of the case (Råheim et al., 2016). This required more “proximity” than we are used to in qualitative research and a distancing from a clinical-reductionist way of looking as well as letting our *inner*standings breathe. In line with the recommendation to justify the selection for the case (Hyett et al., 2014), we want to add that this selection is not only a rational choice (logos) but also informed by emotions (pathos) and morality (ethos). Delving into these emotions and moralities is hard but fulfiling relational “work”.

Another lesson we can draw from this is that secondary analysis of a case through a feminist lens can reveal new and relevant patterns of understanding, and is thus worthwhile. Engaging with the case, relating it to our personal experiences, led to an understanding much deeper than would be the case if there were an element of detachment. This engagement with the case developed the “*inner*standing” of all engaged in the case. So, it became a liberating event for all of us (Abma et al., 2019; Ahmed, 2017; Freire, 1970). We have experienced the importance of connecting our personal lives to the stories of those whose lives we study and increased our understanding of its relevance. We have deepened our understanding of how insight in our own personal experiences, desires, and vulnerabilities offers helpful resources to understand others, and to deconstruct the dichotomy of researcher and researched, patient and doctor. This includes the mutual learning on how much pain and effort it requires to become a “normal” person. How difficult it is to fit into social norms, how we have internalized those norms, and how we constantly measure and discipline ourselves; but also how liberating it can be to accept ourselves as we are, and to develop a more horizontal intrapsychic relationship to our needs and desires (Benjamin, 1988; Kunneman, 2017). Ellen came to the realization that she is not so different from us; that we all struggle to lead a good life. It made her feel less ashamed of her OCD label. This ethical hermeneutic use of case study research has not yet gained much attention, and we recommend it as a fruitful path to further explore in order to generate more depth and impact, and understandings that really matter to people. We can also see that the case has new layers, including a self-reflexive personal and intrapsychic layer as well as a more sociocultural interpretation of structural inequalities related to compulsive behaviour in white middle-class women. These new and dynamically connected intrapsychic and social layers had a de-stigmatizing effect because these illuminated that OCD is not a factual trait of the person or a medical pathology, but rather an extreme response to existential questions in life we all have to deal with.

A related question is whether empathic understanding could turn into over-identification with one perspective. The notion of multiple partiality requires naturalistic case researchers to move among various perspectives, in this case between the perspectives of patient and therapist, and between a feminist and clinical perspective and our own embodied experiences. Such petulance is known as triangulation; to generate meaning by identifying different, and sometimes conflicting ways the case is being seen. Reflexivity, as a posture of reflection during the whole research process, was important to remain aware of the frames that we as researchers used to understand the case (Råheim et al., 2016). During the second analysis, this reflexivity was more targeted at a critical questioning of underlying structural inequalities and its interference with intrapsychic dynamics (Verdonk, 2015), and it was this critical dialogue amongst us in the research team, including Ellen, that generated our critical consciousness (Ahmed, 2017; Freire, 1970). Conscientization refers to the process in which persons become more aware of the sources of their oppression, and we recognize what Paulo Freire said about liberation that nobody liberates nobody, nobody liberates themselves, but that human beings liberate themselves in communion. As one of us reflected:

This case study is a gift to all of us.

## Conclusion

Practitioners and clinicians are increasingly aware of the importance of person-centred care and the importance of personal disposition to their patients. Clinical wisdom is grounded in the intimate knowledge of thousands of cases, one’s upbringing, personal experiences and structural positioning in life. Naturalistic case study research provides a rich understanding of a single case and can aid practitioners in understanding the person with an illness (versus the illness) and the meaning of person-centred care. The naturalistic case study presented shows that studying the particularity of a person as a case can be appropriate for learning and foster personal and mutual understanding among participants and researchers. Moreover, it illustrates that an atypical case can generate rich *inner*standings and offer new perspectives on complex issues when we engage personally with a case, and reflect on our personal motives and structural position in society. Naturalistic case study research appreciates person-centeredness, meaning, complexity, and context of the studied phenomenon. It adds to regular qualitative research that there is an eye for multi-layered understanding and the power of learning from and with the particular patient as a person.
